# *PTGIS* May Be a Predictive Marker for Ovarian Cancer by Regulating Fatty Acid Metabolism

**DOI:** 10.1155/2023/2397728

**Published:** 2023-02-02

**Authors:** Xiaoqing Wu, Wenping Lu, Chaojie Xu, Cuihong Jiang, Weixuan Zhang, Dongni Zhang, Shasha Cui, Zhili Zhuo, Yongjia Cui, Heting Mei, Ya'nan Wang, Chen Li

**Affiliations:** ^1^Department of Oncology, Guang'anmen Hospital, China Academy of Chinese Medical Sciences, Beijing, China; ^2^The Fifth Affiliated Hospital of Zhengzhou University, Zhengzhou University, Zhengzhou, Henan Province, China; ^3^Department of Oncology, Guang'anmen Hospital South Campus, China Academy of Chinese Medical Sciences, Beijing, China; ^4^Department of Oncology, Yueyang Integrated Traditional Chinese and Western Medicine Hospital Affiliated to Shanghai University of Traditional Chinese Medicine, Shanghai, China; ^5^Department of Biology, Chemistry, Pharmacy, Free University of Berlin, Berlin, Germany

## Abstract

**Background:**

Ovarian cancer tends to metastasize to the omentum, which is an organ mainly composed of adipose tissue. Many studies have found that fatty acid metabolism is related to the occurrence and metastasis of cancers. Therefore, it is possible that fatty acid metabolism-related genes (FAMRG) affect the prognosis of ovarian cancer patients.

**Methods:**

First, profiles of ovarian cancer and normal ovarian tissue transcriptomes were acquired from The Cancer Genome Atlas (TCGA) and the Genotype-Tissue Expression (GTEx) databases. A LASSO regression predictive model was developed via the “glmnet” R package. The nomogram was created via the “regplot.” Gene Set Variation Analysis (GSVA), Kyoto Encyclopedia of Genes and Genomes (KEGG), and Gene Ontology (GO) analyses were conducted to determine the FAMRGs' roles. The percentage of immunocyte infiltration was calculated via CIBERSORT. Using “pRRophetic,” the sensitivity of eight regularly used medications and immunotherapy was anticipated.

**Results:**

125 genes were determined as different expression genes (DEGs). Based on RXRA, ECI2, PTGIS, and ACACB, a prognostic model is created and the risk score is calculated. Analyses of univariate and multivariate regressions revealed that the risk score was a distinct prognostic factor (univariate: HR: 2.855, 95% CI: 1.756-4.739, *P* < 0.001; multivariate: HR: 2.943, 95% CI: 1.800-4.812, *P* < 0.001). The nomogram demonstrated that it properly predicted the 1-year survival rate. The expression of memory B molecular units, follicular helper T molecular units, regulatory T molecular units, and M1 macrophages differed remarkably between the groups at high and low risk (*P* < 0.05). Adipocytokine signaling pathways, cancer pathways, and degradation of valine, leucine, and isoleucine vary between high- and low-risk populations. The findings of the GO enrichment revealed that the extracellular matrix and cellular structure were the two most enriched pathways. *PTGIS*, which is an important gene in fatty acid metabolism, was identified as the hub gene. This result was verified in ovarian cancer and ovarian tissues. The connection between the gene and survival was statistically remarkable (*P* = 0.015). The pRRophetic algorithm revealed that the low-risk group was more adaptable to cisplatin, doxorubicin, 5-fluorouracil, and etoposide (*P* < 0.001).

**Conclusion:**

*PTGIS* may be an indicator of prognosis and a possible therapeutic target for the therapy of ovarian cancer patients. The fatty acid metabolism of immune cells may be controlled, which has an indirect effect on cancer cell growth.

## 1. Introduction

Among gynecological malignant cancer, ovarian cancer has the highest death rate. Seventy percent of ovarian cancer are in an advanced stage at the time of diagnosis. It is estimated that 70% of patients who undergo surgery and chemotherapy will still develop peritoneal metastases within two to three years, resulting in intestine blockages and death [1, 2]. A remarkable public health concern for female patients is ovarian cancer; on the basis of the current incidence and treatment scenario, ovarian cancer is a remarkable public health concern for female patients. A peritoneum consists primarily of fat, and its primary function is to store lipids that are energy dense [3]. Due to its anatomical position, peritoneal metastasis is adaptable to intestinal obstruction as it covers the front of the small intestine and colon [4]. It is theoretically possible to extend the patients' survival rate with ovarian cancer if peritoneal metastasis can be prevented or delayed.

A fatty acid is composed of a carboxyl acid group and a hydrocarbon chain with a variety of carbon lengths and degrees of desaturation. A remarkable portion of lipids are composed of these molecules, such as phospholipids, sphingolipids, and triglycerides. Two pathways of fatty acid synthesis are associated with the rapid proliferation of cancerous cells, exogenous uptake, and de novo lipogenesis. Due to their functions in synthesizing the cell membrane, regulating the fluidity of the cell membrane, and acting as second messengers in multiple signaling pathways, fatty acids play a vital role in tumor proliferation and drug resistance. As adipocytes decompose in the peritoneum, free fatty acids, growth factors, and cytokines are produced [5]. Moreover, adipocytes that secrete fatty acids will release a large number of cytokines, such as tumor necrosis factor-*α*, interleukin- (IL-) 6, IL-8, vascular endothelial growth factor, prostaglandins, and leukotrienes, causing continuous inflammation in the local area [6]. Among the characteristics of tumor metastasis is metabolic disorder, and fatty acid metabolism is a key component [7]. Adipose tissue is the primary source of endogenous fatty acids. Some researchers have explored the role of fatty acid-related genes (FAMRGs) in ovarian cancer, hepatocellular carcinoma, and glioma and have reported some results [8–11]. There was also evidence that FAMRGs were closely related to the growth of ovarian cancer both in vivo and in vitro.

Prostaglandin I_2_ synthase (*PTGIS*) is also referred to as *PGIS*. *PTGIS* is one of FAMRG's encoded human prostaglandin I synthases. It can catalyze many reactions involved in drug metabolism and the synthesis of cholesterol, steroids, and other lipids. This endoplasmic reticulum membrane protein catalyzes the conversion of prostaglandin H_2_ to prostacyclin (prostaglandin I_2_), a potent vasodilator and inhibitor of platelet aggregation. The clear and definite function of upregulated *PTGIS* is protecting patients with cardiovascular diseases, such as pulmonary hypertension, heart failure, and hyperlipidemia. An imbalance of prostacyclin and its physiological antagonist thromboxane A2 contributes to the development of myocardial infarction, stroke, and atherosclerosis [12–14]. The development of cancer has been linked to *PTGIS* in recent years, in accordance with a few studies [15]. However, the role and mechanism of *PTGIS* in ovarian cancer are still unclear, and few studies have been conducted.

In present study, a prognostic model and nomogram have been constructed to obtain reliable prognostic information tailored to individual patients [16]. Through the prediction of drug sensitivity, patients' survival times can be extended and drug resistance can be reduced. The information is of great importance to the clinical decision-making of oncologists as well as to the survival time of patients. The screened hub genes were further analyzed by the Gene Set Variation Analysis (GSVA) analysis. It is more appropriate for evaluating path variation in large heterogeneous populations with complex phenotypic characteristics, such as those found in The Cancer Genome Atlas (TCGA) database [17]. It is effective to apply these methods in order to determine whether the hub genes related to fatty acid metabolism are a prognostic factor for ovarian cancer and insights into how fatty acids contribute to prognosis were provided.

## 2. Materials and Methods

### 2.1. The Sources of the Data

A total of 379 transcriptome profiles from ovarian tumor tissues were retrieved from TCGA database (https://portal.gdc.cancer.gov/) on February 24, 2022, and 88 normal ovarian tissue variations from TCGA database and the Genotype-Tissue Expression (GTEx) database (https://gtexportal.org/home/). It was discovered that 130 instances of severe serous ovarian carcinoma with omental metastases were in the Gene Expression Omnibus (GEO) database GSE138866 (https://www.ncbi.nlm.nih.gov/geo/). The clinical data, which included age, gender, grade, overall survival, and survival status, were also obtained from TCGA database.

### 2.2. Comparison of Fatty Acid-Related Gene Expression between Normal Ovarian and Ovarian Tumor Tissues

The keyword “fatty acid” was searched in the GeneCards database (https://www.genecards.org/), and genes with scores of more than 18 were screened. The relevant research literature was searched to supply the above fatty acid-related genes. Finally, 309 FAMRGs were obtained as candidate genes for the study. The “limma” package of the R software was utilized to determine the levels of expression of potential genes in tumor and regular tissue samples. In both regular and cancer tissues, these genes were determined as deferentially expressed genes (DEGs).

### 2.3. Development of FAMRG Prognostic Models

In order to identify genes associated with prognosis in the cross DEGs between TCGA and geo datasets, both “survival” and “survminer” R modules were utilized for Cox regression. The least absolute shrinkage and selection operator (LASSO) Cox regression analysis was utilized to narrow down the genes associated with prognosis, and the GLMNET package was utilized to develop a model of prognosis. TCGA data served as the training set, whereas the GEO data served as the test set. Via the formula Risk score = ∑_*i*=1_^*n*^(*coef*_*i*_ × *expr*_*i*_), the average value of risk was utilized as the dividing line between high-risk and low-risk categories for each sample. The stability of the prognostic model was validated using principal component analysis (PCA) and receiver operating characteristic (ROC) analysis. The R packages “ggplot2” and “timeROC” were used to generate PCA pictures and ROC curves, respectively [18]. Both the training set and the testing set use PCA. If the area under curve (AUC) value is greater than 0.6, the ROC curve indicates that the model is typically correct. The survival analysis in the training set was conducted via the “survival” R package, and a *P* less than 0.05 indicated a remarkable difference in survival rate between the groups at high and low risks. Values of risk and clinical features were evaluated using univariate and multivariate independent prognostic analyses via the “survival” R software package; *P* less than 0.05 indicated statistical significance.

### 2.4. Creating and Validating the Overall Survival (OS) Nomogram

To verify the precision of the prediction model, a nomogram prognosis model was developed and each sample's score of risk in nomogram was determined. The construction was conducted via the “rms” R package, and the visualization was conducted with “regplot.” Clinical features and the nomogram score of risks were included during model development. The ROC curve is used to determine the model's sensitivity and specificity. An AUC greater than 0.60 was established as the believability threshold for models. Clinical variables and nomogram scores of risks were subjected to univariate and multivariate independent prognostic analyses using the Cox regression.

### 2.5. Immune Cells That Infiltrate Cancer Are Defined

A CIBERSORT analysis was utilized to determine the proportion of 21 distinct types of immunocyte infiltration in each sample [19]. To calculate the scores of immune cell infiltration and immune-related biological function for the groups at high and low risks, the R packages “limma” and “reshape2” were used. “ggpubr” was utilized to illustrate the preceding findings.

### 2.6. A Comparison of High-Risk and Low-Risk DEGs in terms of Their Biological Function

The symbols for c2.cp.kegg.v7.4 were obtained from the Molecular Signatures Database (MSigDB, https://www.gsea-msigdb.org/gsea/index.jsp). To investigate the deferentially expressed pathways between high- and low-risk groups, the “GSVA” R software was utilized. “GOplot” and “enrichplot” R programs were used to visualize the findings of the Gene Ontology (GO) analysis, which was conducted via the R package “org.Hs.eg.db” [20]. An analysis of the protein-protein interaction (PPI) network was published on the Search Instrument for the Recovery of Interacting Genes (STRING) v11.0 website (https://cn.string-db.org/). A protein-protein interaction (PPI) analysis was conducted via the Cytoscape v3.7.2 software, and hub genes were screened via the “cytohubba” application. In order to assess these genes, twelve factors will be considered. The genes with the greatest degree of value were determined as hub genes. A survival analysis and a single sample gene set enrichment analysis (ssGSEA) were conducted on the gene with the highest frequency. As part of the Human Protein Atlas (HPA), immunohistochemistry was utilized to confirm the hub gene signature's expression of proteins (https://www.proteinatlas.org).

### 2.7. Prediction of Anticancer Medication Sensitivity

Via the R package “pRRophetic,” the sensitivity of eight commonly used drugs in the therapy of ovarian cancer was assessed, and the risk groups were compared to the sensitivity of each drug. The threshold for statistical significance was set at *P* < 0.001. Via the “ggplot2” R tool, box plots were used to illustrate the predicted sensitivity of each medication. The TIDE website (http://tide.dfci.harvard.edu/) was utilized to predict the immunotherapy response of patients using transcriptomic biomarkers. The higher the score, the more effective the immunotherapy. Via the “limma” package in R, the scores of the groups at high and low risks were contrasted, and the results were displayed via the “ggpubr” package in R.

### 2.8. Statistical Data Analysis

In this study, the gene expression levels of normal tissue and ovarian cancer tissue were compared via the Wilcoxon test. An evaluation of immunological infiltration between subgroups was also conducted via the Wilcoxon test. Overall survival (OS) was compared between subgroups via the log-rank test. A univariate regression analysis was conducted first in order to evaluate the prognostic variables, followed by a multivariate Cox analysis on the statistically remarkable components in order to assess the independent prognostic value of the risk model. All statistical analyses were conducted using R (version 4.1.1).

## 3. Results

### 3.1. Four FAMRGs That Construct Prognostic Models Were Screened

**Figure 1:**
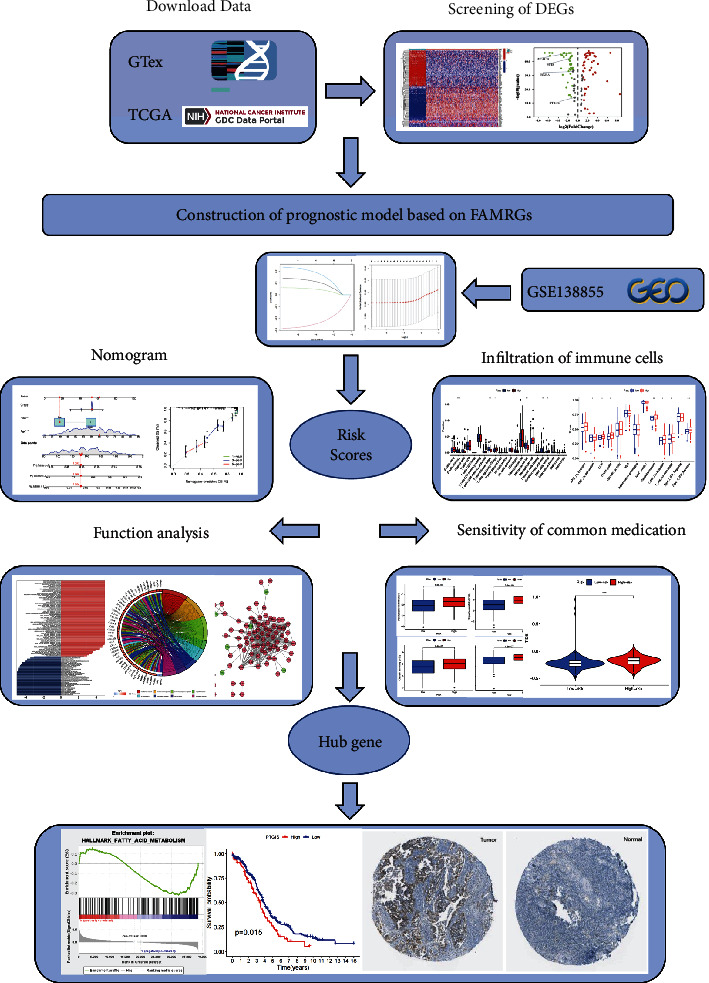
Graphical abstract of the construction of a prognostic index associated with fatty acid metabolism in ovarian cancer.

**Figure 2:**
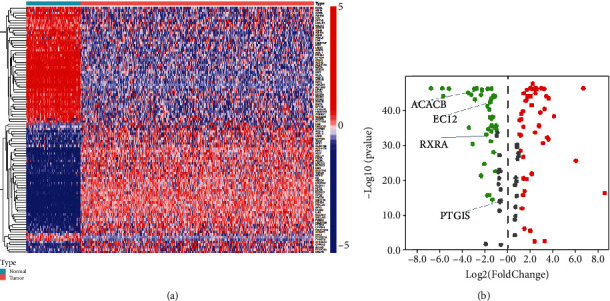
The difference in DEGs between normal ovary tissue and ovarian tumor tissue. (a) Heatmap showing the expression of DEGs. Genes in blue are downregulated. The color red indicates genes that have been upregulated. The volcano plot of DEGs is shown in (b). Green dots represent *P* < 0.05 and FDR < −1.5; red dots represent *P* < 0.05 and FDR > 1.5; grey dots represent *P* < 0.05 and |FDR| < 1.5.

Graphical abstract of the study is shown as Figure 1. To screen the model genes, 376 transcriptome data with matching clinical information were included; 3 transcriptomics without corresponding clinical data are excluded. The clinical characteristics are shown in Table 1. Data in TCGA, GEO, and GTEx databases has been standardized, and batch discrepancies have been resolved. In normal samples, 125 DEGs were determined, of which 59 were downregulated and 66 were upregulated, as indicated in Figures 2(a) and 2(b) and Supplementary Table 1. A total of 100 DEG genes were determined in both TCGA and GEO datasets. Four prognostically associated DEGs were determined using the Cox regression, including RXRA, ECI2, PTGIS, and ACACB, which were also used to develop the prognosis model.

### 3.2. Construction and Verification of Prognosis-Related Model of FAMRGs

**Figure 3:**
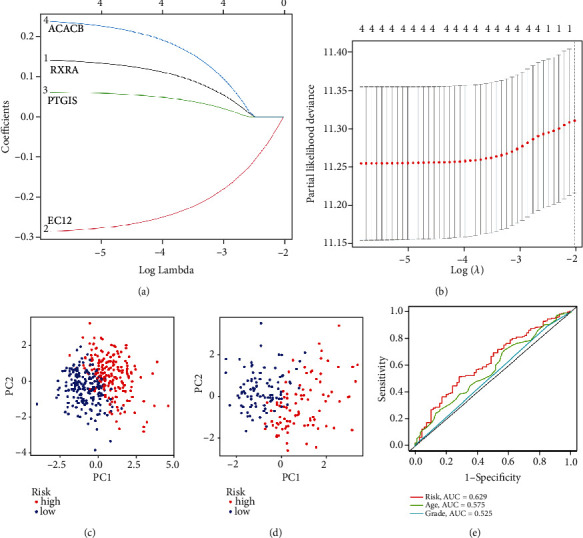
The plot of cross validation and analysis of LASSO regression. (a) The trajectory of the coefficients in the LASSO regression model. (b) The concept behind cross validation. (c) Plot of PCA for FAMRGs in the TCGA database. (d) PCA diagram for FAMRGs in the GEO database. (e) The ROC curve for ovarian tumor risk and clinical characteristics.

While developing the prognostic model, TCGA data were designated as the training set and GEO data as the testing set. Using LASSO regression, the coefficients of the model genes were determined. LASSO regression was utilized to narrow down the model gene coefficients to 0, and cross validation was utilized to identify 0.01 as the minimal and suitable lambda value, as demonstrated in Figures 3(a) and 3(b) and Table 2.

The value of the risks of the training set and testing set samples was obtained by the model. Riskscores = 0.14 × RXRA − 0.28 × ECI2 + 0.06 × PTGIS + 0.24 × ACACB. A PCA was conducted to validate the reliability of the model for risk grouping in the training and testing sets. Figures 3(c) and 3(d) show that the high-risk group and the low-risk group are two separate clusters with obvious decomposition. Although there was confusion at the boundary, the number of samples was mere. The validation results showed that the prognostic model was stable and that the value of risk was reliable. The AUC of the risk score was 0.625, as indicated in Figure 3(e). The ROC curve showed that the risk score has a certain accuracy in predicting prognosis, but age and grade cannot be used to predict prognosis. Univariate and multivariate independent prognostic analyses showed that the risk score was an independent prognostic factor (univariate: HR: 2.855, 95% CI: 1.756-4.739, *P* < 0.001; multivariate: HR: 2.943, 95% CI: 1.800-4.812, *P* < 0.001).

### 3.3. Construction and Validation of Prognostic Nomogram

**Figure 4:**
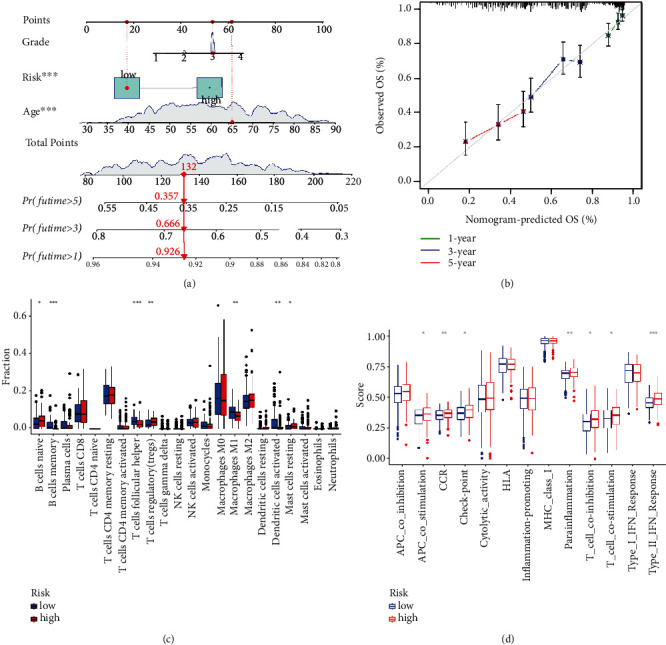
Nomogram prediction model development process. (a) Nomogram of the value of risks and clinical features. (b) Calibration curve of the model for nomogram prediction. (c) The CIBERSORT algorithm's plot of immune cell infiltration and functions between high- and low-risk populations. (d) Immune function distribution between high- and low-risk populations. ^∗^*P* < 0.05;^∗∗^*P* < 0.01; ^∗∗∗^*P* < 0.001.

To further explain and validate the model's stability and explore the clinical significance, a nomogram model with the risk scores was built. As seen in Figure 4(a), the likelihood of surviving one year, three years, and five years diminishes steadily as the overall score increases. The total points may be obtained by adding the points of each variable, and the total points are associated with the likelihood of patients' survival at 1, 3, and 5 years. This result measured the association between the risk scores derived from the prognosis model and survival and may be taken as an additional result of the prognostic model. Moreover, a calibration curve validated the accuracy of the nomogram, as demonstrated in Figure 4(b). The nomogram predicted the 1-year survival rate with the most accuracy, the 3-year survival rate with less accuracy, and the 5-year survival rate with the least accuracy.

### 3.4. Differential Infiltration of Immune Cells in the High-Risk and Low-Risk Groups

The potential function of FAM-related subtypes in ovarian cancer was further analyzed. As indicated in Figure 4(c), CIBERSORT reveals a statistically remarkable difference between the high- and low-risk groups in the expression of 22 immune cells. The proportion of naive B cells, regulatory T cells, and resting mast cells was greater in the high-risk group than in the low-risk group (*P* < 0.05). There was a greater proportion of memory B cells, follicular helper T cells, activated dendritic cells, and M1 macrophages in the low-risk group than that in the high-risk group when *P* < 0.05 was considered. As indicated in Figure 4(d), the scores of chemokine receptor (CCR), parainflammation, and responses to type II IFN were greater in the high-risk group compared to the low-risk group (*P* < 0.05).

### 3.5. DEGs Are Closely Related to the Extracellular Matrix in terms of Their Main Functions

**Figure 5:**
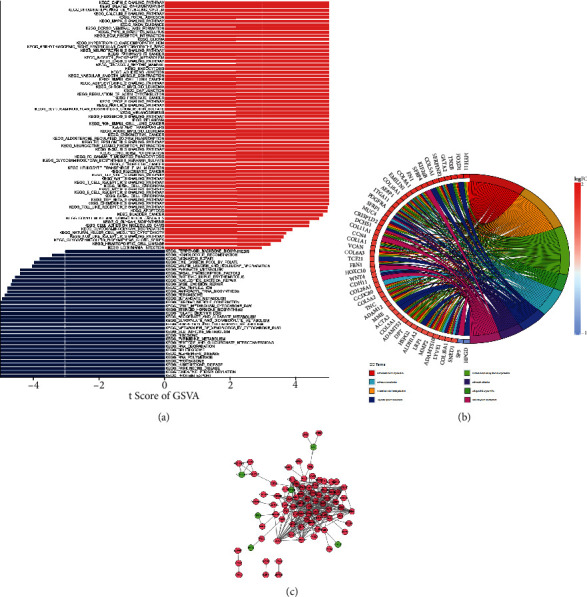
(a) An analysis of the biological role of DEGs. A bar graph illustrates the results of the GSVA. (b) Chordal representation of the GO enrichment analysis. (c) A diagram of the PPI network analysis. Red circles indicate genes that are elevated in tumor tissues. Green circles indicate genes that are downregulated in tumor tissue.

For better studying the potential function of the FAMRG prognosis-related model, the score of each KEGG pathway and GO in ovarian cancer patients was calculated. As indicated in Figure 5(a), the expression of several pathways and gene sets, including adipocytokine signaling pathway, pathways in cancer, and valine leucine and isoleucine degradation, differed between the high- and low-risk groups. In accordance with the GSVA findings, these pathways may be the way via which FAMRGs predicted survival. As depicted in Figure 5(b), extracellular structure organization, cell-substrate adhesion, external encapsulating structure organization, and collagen-containing extracellular matrix were the most abundantly enriched functions in the GO enrichment analysis, which may be the immune mechanism influencing the prognosis of fatty acid genes. PPI network is to reveal the interaction between proteins. As illustrated in Figure 5(c), the PPI network had a total of 86 nodes, 316 edges, and 4 clusters. Four genes (UGT2B17, SST, CRYGB, and THRSP) were downregulated in tumor samples, whereas eighty genes were increased (Supplementary Table 2).

### 3.6. The Difference of Sensitivity of Common Medication in the High- and Low-Risk Groups

**Figure 6:**
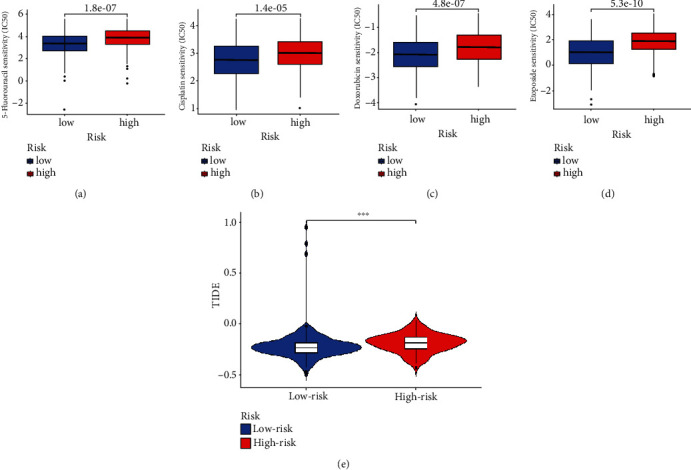
The sensitivity distribution of immunotherapy medicines frequently was used to treat ovarian cancer. (a) The plot of 5-fluororisil sensitivity analysis comparing groups with both minimal and high risk. (b) A cisplatin sensitivity analysis plot comparing groups with low and high risks. (c) The plot of doxorubicin sensitivity analysis compares groups with both minimal and high risk. (d) The plot of etoposide sensitivity analysis comparing groups with both minimal and high risk. (e) The vioplot of TIDE score differential analysis between high- and low-risk groups.

In order to mine the predictive value of the model for drug sensitivity, we conducted pRRophetic algorithm and TIDE evaluation. In accordance with Figures 6(a)–6(d), the pRRophetic algorithm revealed that cisplatin, doxorubicin, 5-fluorouracil, and etoposide were more sensitive in the low-risk group than in the high-risk group. In accordance with Figure 6(e), since the TIDE score increased in the high-risk cohort compared to the low-risk cohort, immunotherapy may be more effective for patients in the high-risk group.

### 3.7. *PTGIS* Performed Crucial Functions in Fatty Acid Metabolism

**Figure 7:**
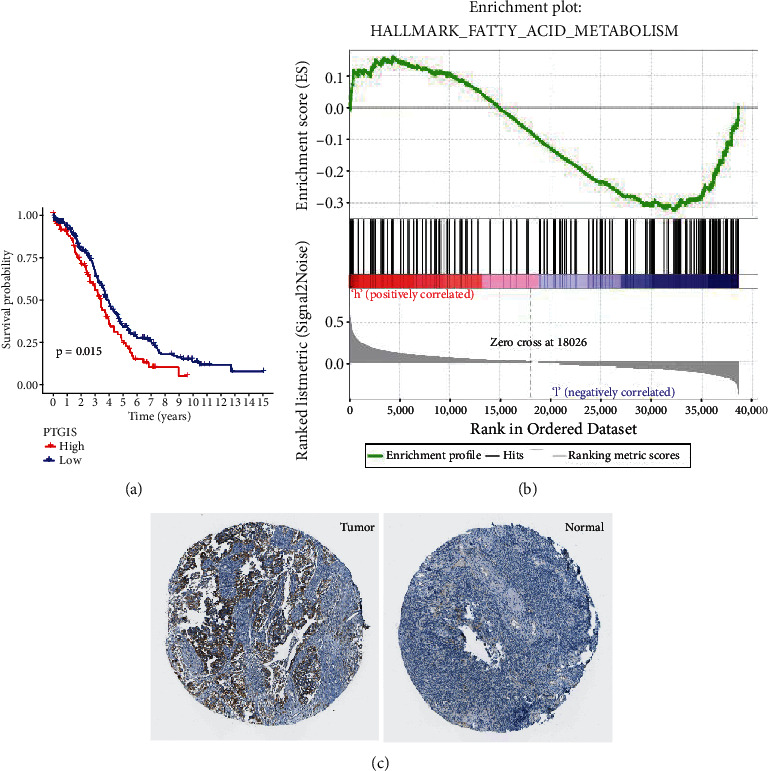
Validation of the hub gene. (a) KM survival analysis of the expression of hub gene *PTGIS.* (b) ssGSEA of PTGIS expression in hallmark database. (c) Immunohistochemistry of PTGIS expression in ovarian cancer and the ovary tissue.

According to the network of PPI, gene *PTGIS* has been maintained as a hub gene. *PTGIS* is deferentially expressed between cancer and normal tissues, and it is an upregulated gene in accordance with logFC > 1. *PTGIS* possesses a centrality score of 0.026, a degree score of 2, a neighborhood connectivity score of 1.5, and a radiality score of 0.602. As indicated in Figure 7(a), survival analysis of TCGA database data revealed that the expression of two genes was positively connected with survival, and the difference was statistically remarkable (*PTGIS* was the hub gene and played an essential role in fatty acid metabolism; *P* = 0.015). The fatty acid metabolism hallmark was enriched in both high expression and low expression of *PTGIS*, as shown in Figure 7(b). In accordance with the HPA database, Figure 7(c) illustrates the protein expression of *PTGIS*. The expression of *PTGIS* was greater in ovarian tumorous tissue as compared to healthy ovarian tissues.

## 4. Discussion

In the study, FAMRGs were found to be deferentially expressed in ovarian cancer and normal ovaries. A prognostic prediction model was constructed and validated based on *RXRA*, *ECI2*, *PTGIS*, and *ACACB*. The results of PCA and ROC curves indicated that the model was accurate and stable. A nomogram model was developed to quantify the connection between the risk scores obtained by the prognostic model and survival. In terms of predicting 1-year survival, the nomogram was more accurate. It was found that there was a greater difference in humoral immune-related cells in the low-risk and high-risk groups in the analysis of immune infiltration. It was mainly the structure of the extracellular matrix and biological processes within the extracellular matrix that were functionally annotated for these genes. *PTGIS*, as a hub gene in the network of genes, played a central role in the process. Sorafenib and immunotherapy were more effective in treating patients in the high-risk group, in accordance with a sensitivity analysis of immunotherapeutic drugs commonly used in the therapy of ovarian cancer.

In accordance with previous studies, fatty acid-related genes are linked with a bad prognosis and high levels of expression in ovarian cancer [8, 10]. Several studies have demonstrated antitumor effects when fatty acid synthase is inhibited [21, 22]. The similar findings were showed in other tumors. The poor prognosis of glioma patients was associated with genetic changes in lipid metabolism [9, 23]. These results were consistent with those reported in the study.

*PTGIS* as one of fatty acid-related genes, patients whose breast cancer tissues express high levels of PGIS have a lower 10-year survival rate. *PTGIS* transiently transfected into MCF-7 cells increased cell viability by 50% [24]. High expression of *PTGIS* could promote the infiltration of tumor-associated macrophages and Tregs in the tumor microenvironment and deteriorate outcomes of patients with lung, ovarian, and gastric cancers [25]. These findings suggest that *PTGIS* could be taken as a potential biomarker of prognosis and tumor-infiltrating immune cells. The *PTGIS* enzyme works in conjunction with the inducible cyclooxygenase-2 enzyme (COX-2) as an upstream enzyme to produce PGI_2_ [26]. PGI_2_, as a PPAR ligand, activated the PPAR signaling pathway. The *PPAR* gene is primarily expressed in adipose tissue. Through direct activation of *PPAR* genes, it regulates adipocyte differentiation, lipid metabolism, and secretion [27]. In this GSEA, we found that *PTGIS* was not enriched at the high expression site as expected, but there were two enrichment peaks, respectively, at the high expression site and the low expression site. This may be due to the different roles of the gene in different diseases; many scholars have found its different roles in different diseases [28–35]. *PTGIS* plays a positive role in cardiovascular diseases by regulating fatty acid metabolism [29, 33, 35, 36], while it is the opposite in cancer [25, 37–42]. This phenomenon should be related to the metabolic disorder of cancer cells, but the specific mechanism is still unclear.

In immune cells, it also regulates the differentiation and polarization of macrophages and controls lipid metabolism through the regulation of genes such as *CD36*, *FABP4*, *LXRA* and *PGAR* in monocytes, macrophages, and dendritic cells. By activating PPAR, macrophages were transformed into alternative M2 macrophages [43]. The phenomenon was likewise validated by the findings. In the research, CIBERSORT revealed substantial differences between the high- and low-risk groups in terms of cells of naive memory B, follicular helper, regulatory T, M1 macrophages, activated dendritic, and resting mast cells. The low-risk group had more infiltration of M1 macrophages, B cells, T cells, and dendritic cells than the high-risk group. Through the traditional PPAR transactivation, *PTGIS* may affect the lipid transport, metabolism, and presentation of immune cells.

In the examination of medication sensitivity, the samples from the low-risk category showed greater sensitivity to the most widely used chemotherapy. This result has been extensively documented in in vivo and in vitro research. In 2800 women with breast cancer, the benefit of getting docetaxel was reduced for obese patients than for normal-weight people [44]. Adipocyte-mediated metabolism decreases the concentration of active daunorubicin [45]. Fatty acids are essential components of the cell membrane and serve an essential function in maintaining its fluidity. The greater the membrane fluidity, the simpler it is for malignant cells to spread. The majority of adipogenesis in malignant cells involves the production of saturated and monounsaturated fatty acids that are more stable. These fatty acids are not adaptable to the oxidative stress generated by chemotherapy medications (such as Adriamycin) and protect cancer cells from death [46]. The development of multidrug-resistant cancers may be connected to the rise in membrane rigidity. Thus, the permeability of anticancer medications is poor [47]. The effectiveness of checkpoint inhibitors seems to be greater in individuals with fatty acid metabolic abnormalities. As compared to patients with a normal BMI, obesity is associated with improved progression-free survival and overall survival in male patients receiving targeted treatment or immunotherapy [48]. Research demonstrated that a high BMI was associated with better outcomes with targeted treatments, and changes in fatty acid metabolism were associated with better outcomes with checkpoint inhibitors [49, 50].

Some preclinical investigations suggest that fatty acid metabolism might be a viable therapeutic target. A mouse model of human cavity carcinoma in situ is shown to inhibit tumor growth and spread [51]. Engineering fatty acid Pt (IV) prodrugs demonstrate fatty acid consumption as well as the development of cisplatin-resistant ovarian cancer [52]. BMS309403, a competitive inhibitor of endogenous fatty acid binding, greatly reduced adipocyte-mediated omental metastasis in ovarian tumor cells [5, 53]. These findings show fatty acid metabolism is a potential treatment alternative for ovarian cancer that warrants further investigation.

It has been shown in numerous studies that cancers other than ovarian cancer are associated with genes involved in fatty acid metabolism. But there is no doubt that the prognosis of patients with ovarian cancer is more frequently associated with fatty acid metabolism (most ovarian tumor metastases are located in the omentum, which consists mainly of adipose tissue). Research has demonstrated that FARMGs can be used to predict the prognosis of ovarian cancer. Unlike previous studies, this study examines the potential influence of fatty acid metabolism on ovarian cancer as well as its effect on the immune system. A metabolic imbalance of fatty acids may affect prognosis not only by promoting cancer cell proliferation but also by modulating the immunological microenvironment.

Little research has investigated the possible involvement of *PTGIS* in ovarian cancer, which is one of the study's strengths. This work may provide fresh insight into the search for prognostic indicators and the investigation of therapeutic strategies for ovarian cancer. Another feature is the research design. Based on FAMRGs, a prognosis model and nomogram were built; risk, clinical features, and prognosis were quantified; and clinical decision-making evidence was presented. The model has undergone exhaustive internal and external validation to assure its legitimacy.

There are a few limitations to the study. In vivo and in vitro confirmation of the results is required. Insufficient investigation has been conducted regarding downstream access. This is also the direction in which the work will be directed in the future.

## 5. Conclusion

It may be advantageous to offer more aggressive treatment to patients at high risk, to combine chemotherapy with checkpoint inhibitors whenever possible, and to avoid prescribing chemotherapy solely based on study results. The future study will examine the relationship between PTGIS and lipid metabolism molecules such as PPAR, FABP4, FASN, and CD36, as well as the effect of fatty acid metabolism on the growth and invasion of ovarian tumorous cells.

Finally, *PTGIS* may serve as a prognostic indicator and therapeutic target for patients with ovarian cancer. By modulating the breakdown of fatty acids in cancerous cells and immune cells, PTGIS inhibits the multiplication of malignant cells in both a direct and indirect manner.

## Figures and Tables

**Table 1:** Clinicopathological characteristics of OV samples from TCGA and GEO databases.

Characteristics	TCGA-OV cohort, *N* = 376	GSE138866 cohort, *N* = 130
Age		
≤65	259 (68.88%)	73 (56.15%)
>65	117 (31.12%)	57 (43.85%)
Gender		
Female	376 (100.00%)	130 (100%)
Stage		
II-III	0	100 (76.92%)
IV	0	21 (16.15%)
Unknown	376 (100.00%)	9 (6.92%)
Grade		
G1	1 (0.27%)	0
G2	44 (11.70%)	0
G3	322 (85.64%)	123 (94.62%)
G4	1 (0.27%)	0
Unknown	8 (2.13%)	7 (5.38%)
Survival status		
Alive	146 (38.52%)	19 (14.62%)
Dead	230 (60.69%)	111 (85.38%)
Unknown	0	0
The median follow-up time (year)	2.81	2.81

**Table 2:** Prognostic model based on four genes.

Gene name	Coef
RXRA	0.14
ECI2	-0.28
PTGIS	0.06
ACACB	0.24

## Data Availability

The datasets analyzed during the current study are available in TCGA database (https://portal.gdc.cancer.gov/), GEO database (https://www.ncbi.nlm.nih.gov/geo/), GTex database (https://gtexportal.org/home/), STRING v11.0 (https://string-db.org/), and the Human Protein Atlas (https://www.proteinatlas.org/).

## Supplementary Materials

Supplementary 1 Supplementary Table 1: screening for prognostically relevant DEGs.

Supplementary 2 Supplementary Table 2: protein interactions in PPI networks.
