# Supplementary material for: PTGIS May Be a Predictive Marker for Ovarian Cancer by Regulating Fatty Acid Metabolism
Source: Comput Math Methods Med. 2023 Feb 2;2023:2397728. doi: 10.1155/2023/2397728 (PMC9918844; doi:10.1155/2023/2397728)
Supplement: Supplementary 1 — Supplementary Table 1: screening for prognostically relevant DEGs. [file 2397728.f1.docx]

Supplementary table 2 Protein interactions in PPI networks

| Gene | Type |
| --- | --- |
| ACTA2 | up |
| EEF1A2 | up |
| MMP2 | up |
| SERPINE1 | up |
| COL1A1 | up |
| PDGFRA | up |
| CDH11 | up |
| COL3A1 | up |
| FBN1 | up |
| FN1 | up |
| LMOD1 | up |
| COL5A1 | up |
| COL5A2 | up |
| MYH11 | up |
| ACTG2 | up |
| ADAM12 | up |
| TIMP3 | up |
| COL6A3 | up |
| ADAMTS10 | up |
| ADAMTS2 | up |
| VCAN | up |
| THBS2 | up |
| COL16A1 | up |
| COL8A1 | up |
| TNXB | up |
| ZNF469 | up |
| COL11A1 | up |
| ADH1B | up |
| ALDH1A2 | up |
| ALDH1A3 | up |
| UGT2B17 | down |
| AEBP1 | up |
| TCF21 | up |
| AOC3 | up |
| MRC1 | up |
| ARX | up |
| SST | down |
| BARX1 | up |
| SFRP1 | up |
| CACNA1H | up |
| SCNN1G | up |
| CCDC80 | up |
| OGN | up |
| CXCL12 | up |
| MOXD1 | up |
| DCHS1 | up |
| CILP | up |
| DPT | up |
| SFRP4 | up |
| COL10A1 | up |
| COL26A1 | up |
| ITGA11 | up |
| HSPG2 | up |
| WNT4 | up |
| FABP4 | up |
| CRYGB | down |
| FNDC1 | up |
| TNC | up |
| LYVE1 | up |
| EGR3 | up |
| NR4A3 | up |
| EMILIN1 | up |
| F13A1 | up |
| THRSP | down |
| PLIN4 | up |
| NGFR | up |
| LRP1 | up |
| MUC5B | up |
| LRRC4B | up |
| FOXD1 | up |
| GFRA1 | up |
| HIC1 | up |
| HPGD | down |
| PTGIS | up |
| LEFTY1 | up |
| TFCP2L1 | up |
| MME | up |
| LYNX1 | up |
| SLURP1 | up |
| LYPD2 | up |
| MUC4 | up |
| TFF2 | down |
| TNNT3 | up |
| SORCS2 | up |
| PODN | up |
| SNED1 | up |
