# Supplementary material for: PTGIS May Be a Predictive Marker for Ovarian Cancer by Regulating Fatty Acid Metabolism
Source: Comput Math Methods Med. 2023 Feb 2;2023:2397728. doi: 10.1155/2023/2397728 (PMC9918844; doi:10.1155/2023/2397728)
Supplement: Supplementary 2 — Supplementary Table 2: protein interactions in PPI networks. [file 2397728.f2.docx]

| gene | conMean | treatMean | logFC | pValue | fdr |
| --- | --- | --- | --- | --- | --- |
| ALDH2 | 49.64 | 6.36 | -2.96 | <0.001 | <0.001 |
| ACSBG1 | 0.26 | 0.08 | -1.7 | <0.001 | <0.001 |
| HSD17B3 | 0.74 | 0.31 | -1.24 | <0.001 | <0.001 |
| PTGES | 0.85 | 5.82 | 2.78 | <0.001 | <0.001 |
| PDHA1 | 40.2 | 15.06 | -1.42 | <0.001 | <0.001 |
| ACSF3 | 5.25 | 1.96 | -1.42 | <0.001 | <0.001 |
| MAOA | 14.06 | 1.9 | -2.88 | <0.001 | <0.001 |
| ALOX15B | 0.59 | 1.62 | 1.46 | <0.001 | <0.001 |
| PPT2 | 18.4 | 3.85 | -2.26 | <0.001 | <0.001 |
| GPX1 | 59.65 | 278.25 | 2.22 | <0.001 | <0.001 |
| ENO3 | 4.31 | 1.63 | -1.4 | <0.001 | <0.001 |
| ACBD4 | 8.56 | 3.83 | -1.16 | <0.001 | <0.001 |
| CA6 | 0.01 | 0.04 | 2.37 | 0.003 | 0.003 |
| CYP4F8 | 0.03 | 0.02 | -0.59 | 0.024 | 0.026 |
| ACBD6 | 11.92 | 5.37 | -1.15 | <0.001 | <0.001 |
| UBE2L6 | 14.15 | 62.16 | 2.14 | <0.001 | <0.001 |
| CYP2C9 | 0.05 | 0.02 | -1.35 | <0.001 | <0.001 |
| ADH1A | 0.22 | 0.02 | -3.44 | <0.001 | <0.001 |
| DPEP3 | 0.04 | 15.07 | 8.56 | <0.001 | <0.001 |
| ALOXE3 | 0.1 | 0.45 | 2.12 | <0.001 | <0.001 |
| SLC25A1 | 13.57 | 41.42 | 1.61 | <0.001 | <0.001 |
| ALDH1A1 | 71.5 | 7.7 | -3.21 | <0.001 | <0.001 |
| CA4 | 1.9 | 0.52 | -1.88 | <0.001 | <0.001 |
| ABCD1 | 1.6 | 8.65 | 2.43 | <0.001 | <0.001 |
| GLUL | 268.62 | 40.12 | -2.74 | <0.001 | <0.001 |
| ADH5 | 45.27 | 15.92 | -1.51 | <0.001 | <0.001 |
| HACD2 | 2.43 | 13.8 | 2.5 | <0.001 | <0.001 |
| OSTC | 23.47 | 44.86 | 0.93 | <0.001 | <0.001 |
| CYP1B1 | 1.8 | 4.58 | 1.35 | <0.001 | <0.001 |
| HMGCS2 | 0.11 | 1.1 | 3.26 | 0.002 | 0.003 |
| CPOX | 3.06 | 5.41 | 0.82 | <0.001 | <0.001 |
| ACSM6 | 0.01 | 0.03 | 1.46 | <0.001 | <0.001 |
| FAAH | 2.73 | 5.94 | 1.12 | <0.001 | <0.001 |
| NTHL1 | 5.76 | 10.01 | 0.8 | <0.001 | <0.001 |
| SDHD | 21.32 | 40.6 | 0.93 | <0.001 | <0.001 |
| ALDH1B1 | 2.94 | 12.7 | 2.11 | <0.001 | <0.001 |
| ADH4 | 0.14 | 0.03 | -2.34 | <0.001 | <0.001 |
| UROD | 32.69 | 16.2 | -1.01 | <0.001 | <0.001 |
| ABCC1 | 2.82 | 9.12 | 1.69 | <0.001 | <0.001 |
| DHCR24 | 11.49 | 138.29 | 3.59 | <0.001 | <0.001 |
| HCCS | 2.33 | 9.22 | 1.99 | <0.001 | <0.001 |
| RXRA | 9.37 | 4.76 | -0.98 | <0.001 | <0.001 |
| ACOXL | 0.05 | 0.3 | 2.44 | <0.001 | <0.001 |
| NDUFAB1 | 16.8 | 34.86 | 1.05 | <0.001 | <0.001 |
| PCTP | 2.36 | 3.82 | 0.7 | <0.001 | <0.001 |
| THEM5 | 0.14 | 1.03 | 2.86 | <0.001 | <0.001 |
| CPT1B | 2.84 | 0.56 | -2.35 | <0.001 | <0.001 |
| LTA4H | 41.89 | 14 | -1.58 | <0.001 | <0.001 |
| CYP8B1 | 0.04 | 0.16 | 2 | <0.001 | <0.001 |
| HSD17B8 | 2.48 | 13.58 | 2.45 | <0.001 | <0.001 |
| SUCLG2 | 6.41 | 16.83 | 1.39 | <0.001 | <0.001 |
| CYP1A2 | 0.01 | 0.43 | 6.01 | <0.001 | <0.001 |
| ELOVL3 | 0.11 | 1.9 | 4.07 | <0.001 | <0.001 |
| TDO2 | 0.15 | 1.73 | 3.49 | <0.001 | <0.001 |
| ACADS | 3.3 | 5.52 | 0.74 | <0.001 | <0.001 |
| SCD5 | 23.01 | 13.97 | -0.72 | <0.001 | <0.001 |
| GGT5 | 9.27 | 2.65 | -1.8 | <0.001 | <0.001 |
| TBXAS1 | 1.04 | 1.6 | 0.63 | <0.001 | <0.001 |
| AMACR | 1.35 | 0.8 | -0.75 | <0.001 | <0.001 |
| PCCA | 6.03 | 2.5 | -1.27 | <0.001 | <0.001 |
| ACSF2 | 17.48 | 6.47 | -1.43 | <0.001 | <0.001 |
| ALDH3A1 | 0.63 | 1.5 | 1.25 | <0.001 | <0.001 |
| GPX2 | 1.29 | 0.34 | -1.94 | 0.018 | 0.02 |
| CYP4F3 | 0.13 | 1.13 | 3.12 | <0.001 | <0.001 |
| MAPKAPK2 | 14.62 | 22.95 | 0.65 | <0.001 | <0.001 |
| ALAD | 11.59 | 7.37 | -0.65 | <0.001 | <0.001 |
| CYP2C19 | 0.01 | 0 | -2.06 | <0.001 | <0.001 |
| LDHA | 82.18 | 132.61 | 0.69 | <0.001 | <0.001 |
| ADH6 | 0.39 | 0.14 | -1.49 | <0.001 | <0.001 |
| ACAT1 | 19.29 | 5.65 | -1.77 | <0.001 | <0.001 |
| ELOVL1 | 10.36 | 35.69 | 1.78 | <0.001 | <0.001 |
| FAAH2 | 0.52 | 4.75 | 3.2 | <0.001 | <0.001 |
| ECHS1 | 18.5 | 75.98 | 2.04 | <0.001 | <0.001 |
| IDH3B | 57.36 | 37.53 | -0.61 | <0.001 | <0.001 |
| FH | 7.45 | 24.43 | 1.71 | <0.001 | <0.001 |
| ETFDH | 5.99 | 2.52 | -1.25 | <0.001 | <0.001 |
| AC1SM3 | 109.84 | 3.02 | -5.19 | <0.001 | <0.001 |
| PLA2G4A | 1.22 | 3.95 | 1.69 | <0.001 | <0.001 |
| D2HGDH | 13.81 | 5.22 | -1.4 | <0.001 | <0.001 |
| SLC22A5 | 2.77 | 1.49 | -0.9 | <0.001 | <0.001 |
| XIST | 170.06 | 3.08 | -5.79 | <0.001 | <0.001 |
| NUDT7 | 2.11 | 1.25 | -0.76 | <0.001 | <0.001 |
| VNN1 | 0.13 | 1.54 | 3.57 | <0.001 | <0.001 |
| ACO2 | 9.21 | 16.87 | 0.87 | <0.001 | <0.001 |
| UGDH | 5.52 | 9.77 | 0.82 | <0.001 | <0.001 |
| MDH2 | 30.03 | 81.11 | 1.43 | <0.001 | <0.001 |
| PPT1 | 12.1 | 54.95 | 2.18 | <0.001 | <0.001 |
| EPHX2 | 12.34 | 4.04 | -1.61 | <0.001 | <0.001 |
| ACOT6 | 0.17 | 0.06 | -1.62 | <0.001 | <0.001 |
| ACOT2 | 4.66 | 7.62 | 0.71 | <0.001 | <0.001 |
| SLC27A3 | 11.87 | 6.66 | -0.83 | <0.001 | <0.001 |
| SERINC1 | 43.71 | 28.98 | -0.59 | <0.001 | <0.001 |
| HSD17B10 | 25.66 | 56.94 | 1.15 | <0.001 | <0.001 |
| ACLY | 6.5 | 16.62 | 1.35 | <0.001 | <0.001 |
| ECI2 | 20.96 | 7.2 | -1.54 | <0.001 | <0.001 |
| APEX1 | 63.43 | 134.17 | 1.08 | <0.001 | <0.001 |
| NUDT19 | 1.09 | 8.59 | 2.98 | <0.001 | <0.001 |
| PCCB | 3.25 | 7.48 | 1.2 | <0.001 | <0.001 |
| ACSL5 | 3.44 | 9.01 | 1.39 | <0.001 | <0.001 |
| PRXL2B | 2.48 | 14.74 | 2.57 | <0.001 | <0.001 |
| ACSL6 | 0.13 | 0.07 | -0.83 | <0.001 | <0.001 |
| ACSL4 | 3.65 | 9.52 | 1.38 | <0.001 | <0.001 |
| IL4I1 | 0.29 | 29.59 | 6.67 | <0.001 | <0.001 |
| PTGIS | 12.25 | 7.46 | -0.72 | <0.001 | <0.001 |
| ACAD11 | 12.32 | 0.11 | -6.78 | <0.001 | <0.001 |
| INMT | 6.55 | 0.76 | -3.1 | <0.001 | <0.001 |
| ACACB | 6.49 | 1.35 | -2.27 | <0.001 | <0.001 |
| CPT1C | 4.2 | 1.95 | -1.11 | <0.001 | <0.001 |
| ADH1B | 61.56 | 1.19 | -5.7 | <0.001 | <0.001 |
| ACOX2 | 8.02 | 2.32 | -1.79 | <0.001 | <0.001 |
| DECR1 | 20.3 | 10.01 | -1.02 | <0.001 | <0.001 |
| LTC4S | 3.23 | 0.29 | -3.49 | <0.001 | <0.001 |
| ELOVL7 | 0.24 | 2.52 | 3.39 | <0.001 | <0.001 |
| NBN | 3.59 | 8.31 | 1.21 | <0.001 | <0.001 |
| LGALS1 | 321.87 | 192.84 | -0.74 | <0.001 | <0.001 |
| MGLL | 1.81 | 4.68 | 1.37 | <0.001 | <0.001 |
| SLC27A2 | 0.11 | 1.04 | 3.24 | <0.001 | <0.001 |
| MLYCD | 1.3 | 0.83 | -0.65 | <0.001 | <0.001 |
| PCBD1 | 19.25 | 79.38 | 2.04 | <0.001 | <0.001 |
| SLC25A17 | 9.03 | 5.51 | -0.71 | <0.001 | <0.001 |
| DBI | 37.26 | 23.33 | -0.68 | <0.001 | <0.001 |
| CPT2 | 1.87 | 7.63 | 2.03 | <0.001 | <0.001 |
| GPD2 | 1.95 | 10.28 | 2.39 | <0.001 | <0.001 |
| HSD17B7 | 5.55 | 2.23 | -1.31 | <0.001 | <0.001 |
| ACOT1 | 1.21 | 1.88 | 0.64 | <0.001 | <0.001 |

Supplementary table 1 Screening for prognostically relevant DEGs
